# Usage patterns, health, and nutritional status of long-term multiple dietary supplement users: a cross-sectional study

**DOI:** 10.1186/1475-2891-6-30

**Published:** 2007-10-24

**Authors:** Gladys Block, Christopher D Jensen, Edward P Norkus, Tapashi B Dalvi, Les G Wong, Jamie F McManus, Mark L Hudes

**Affiliations:** 1 School of Public Health, 50 University Hall, University of California, Berkeley, CA, 94720, USA; 2 Department of Medical Research, Our Lady of Mercy Medical Center, 600 East 233^rd ^Street, Bronx, NY, 10466, USA; 3 Shaklee Corporation, 4747 Willow Road, Pleasanton, CA, 94588, USA; 4 Nutritional Sciences and Toxicology, 234 Morgan Hall, University of California, Berkeley, CA, 94720, USA

## Abstract

**Background:**

Dietary supplement use in the United States is prevalent and represents an important source of nutrition. However, little is known about individuals who routinely consume multiple dietary supplements. This study describes the dietary supplement usage patterns, health, and nutritional status of long-term multiple dietary supplement users, and where possible makes comparisons to non-users and multivitamin/mineral supplement users.

**Methods:**

Using a cross-sectional study design, information was obtained by online questionnaires and physical examination (fasting blood, blood pressure, body weight) from a convenience sample of long-term users of multiple dietary supplements manufactured by Shaklee Corporation (Multiple Supp users, n = 278). Data for non-users (No Supp users, n = 602) and multivitamin/mineral supplement users (Single Supp users, n = 176) were obtained from the National Health and Nutrition Examination Survey (NHANES) 2001–2002 and NHANES III 1988–1994. Logistic regression methods were used to estimate odds ratios with 95% confidence intervals.

**Results:**

Dietary supplements consumed on a daily basis by more than 50% of Multiple Supp users included a multivitamin/mineral, B-complex, vitamin C, carotenoids, vitamin E, calcium with vitamin D, omega-3 fatty acids, flavonoids, lecithin, alfalfa, coenzyme Q10 with resveratrol, glucosamine, and a herbal immune supplement. The majority of women also consumed gamma linolenic acid and a probiotic supplement, whereas men also consumed zinc, garlic, saw palmetto, and a soy protein supplement. Serum nutrient concentrations generally increased with increasing dietary supplement use. After adjustment for age, gender, income, education and body mass index, greater degree of supplement use was associated with more favorable concentrations of serum homocysteine, C-reactive protein, high-density lipoprotein cholesterol, and triglycerides, as well as lower risk of prevalent elevated blood pressure and diabetes.

**Conclusion:**

This group of long-term multiple dietary supplement users consumed a broad array of vitamin/mineral, herbal, and condition-specific dietary supplements on a daily basis. They were more likely to have optimal concentrations of chronic disease-related biomarkers, and less likely to have suboptimal blood nutrient concentrations, elevated blood pressure, and diabetes compared to non-users and multivitamin/mineral users. These findings should be confirmed by studying the dietary supplement usage patterns, health, and nutritional status of other groups of heavy users of dietary supplements.

## Background

Diet and nutrition play important roles in the maintenance of health and prevention of disease [1,2]. Dietary supplements represent an important source of essential nutrients since they are widely used and often contain 100% or more of the Daily Value of one or more nutrients [3,4]. They are also of concern because of potential adverse effects [5]. Prior studies have reported the prevalence of dietary supplement usage and the characteristics of users in the United States (US) population. In the most recent reporting of nationwide survey data, the National Health and Nutrition Examination Survey (NHANES) 1999–2000, 52% of adults reported taking a dietary supplement in the past 30 days [4]. This and other surveys have generally found that dietary supplement usage is more common in women than men, in older participants than in younger ones, in Whites compared with Blacks and Mexican Americans, in the more educated as compared to the less educated, and in the more affluent as compared to less affluent [4]. In NHANES 1999–2000, approximately 47% of dietary supplement users reported taking only one type of supplement (most commonly a multivitamin/mineral) [4]. Only 3 individuals out of over 11,000 surveyed reported taking 20 or more different supplements in the past 30 days. Consequently, little if any descriptive information has been reported about individuals who typically consume multiple dietary supplements.

This cross-sectional study was undertaken to describe the dietary supplement usage patterns, serum nutrient and biomarker concentrations, and health status of a convenience sample of individuals who were daily users of multiple dietary supplements (median of 26 different dietary supplements taken daily in the prior 12 months). In addition, biomarker concentrations and the health status of multiple dietary supplement users were compared with two other convenience samples assembled from NHANES: non-users of supplements and those who consumed a multivitamin/mineral supplement only.

## Methods

### Participants

To assemble a sample of long-term users of multiple dietary supplements, individuals who had been consumers of dietary supplements for ≥ 20 years from a dietary supplement manufacturer and distributor (Shaklee Corporation, Pleasanton, CA) were invited to participate in the study. The invitation was sent by electronic mail to approximately 1,200 individuals meeting this criterion. Of the total invitees, 435 successfully completed online questionnaires to obtain information about height, annual household income, education, medical history and current medical conditions, and dietary supplement usage. Reasons for non-participation were not investigated. A subset of 300 individuals who completed the questionnaires and were free of cancer, other than non-melanoma skin cancer, were randomly chosen and asked to attend a physical examination for the purpose of providing a 12-hour fasting blood sample and having their body weight and blood pressure measured. A final analytic sample of 278 participants had complete questionnaire and examination data. These individuals composed the 'Multiple Supp' users. The study was reviewed and approved by an independent institutional review board (Health Research and Studies Center, Palo Alto, California), and all participants provided informed consent.

### Data collection

For the Multiple Supp users, online questionnaires were used to obtain information about height, annual household income, education, medical history and current medical conditions, and current dietary supplement usage. Questions about medical history and current medical conditions were patterned after questions asked in NHANES 2001–2002 [6]. The dietary supplement questionnaire included 57 dietary supplements and foods sold by Shaklee in 2005 and asked about usual intake in the previous 12 months. The questionnaire included four intake frequency categories including 'rarely or never used,' '1–3 days per week,' '4–6 days per week,' and 'every day,' and also asked about usual serving size.

Physical examinations were conducted during a national meeting of Shaklee product consumers held in Chicago, Illinois in August 2005. Data collection took place between July and August of 2005. Body weight was measured using a physician's scale with shoes and heavy clothing removed. Triplicate blood pressure measurements were taken by an automated Welch Allyn device (Model 5201) with an appropriate-sized cuff after subjects were seated quietly in a chair for 2 minutes, with feet on the ground and with the arm at the level of the heart. Twelve-hour fasting blood samples were collected, processed, aliquoted and stored frozen at -80°C. Samples were assayed for red blood cell (RBC) folate, serum ascorbic acid stabilized using 10% metaphosphoric acid, alpha tocopherol, carotenoids, retinol, 25-hydroxyvitamin D, ferritin, C-reactive protein (CRP), lipids, and homocysteine (Table 1). Assay procedures followed those that were utilized in NHANES 2001–2002 and in NHANES III [6-12] as described in Table 1.

**Table 1:** Micronutrient assay methods.

Analyte	Method	Ref.
RBC folate (nmol/L)	Quantaphase Folate Radioassay Kit, Bio-Rad Laboratories	7
Retinol (μg/dL)	Isocratic reversed-phase HPLC with UV/Vis detection	8
Ascorbic acid (mg/dL)	Isocratic reversed-phase HPLC with Electrochemical detection*	7
Alpha tocopherol (mg/dL)	Isocratic reversed-phase HPLC with UV/Vis detection	8
Alpha carotene (μg/dL)	Isocratic reversed-phase HPLC with UV/Vis detection	8
Beta carotene (μg/dL)	Isocratic reversed-phase HPLC with UV/Vis detection	8
Ferritin (μg/L)	Quantlmune Ferritin IRMA kit, Bio-Rad Laboratories	7
25-hydroxyvitamin D (nmol/L)	Quantlmune Ferritin IRMA kit, Bio-Rad Laboratories	7
Homocysteine (μmol/L)	Isocratic reversed-phase HPLC with Fluorescence detection	9
C-reactive protein (mg/L)	Latex-enhanced nephelometry, Behring Nephelometer Analyzer for NHANES assays and Hitachi 917 Analyzer for Multiple Supp user assays	7
Total cholesterol (mg/dL)	Timed endpoint, coupled enzymatic methodology	10
LDL-cholesterol (mg/dL)	Calculated**	7
HDL-cholesterol (mg/dL)	Polyanion/divalent cation precipitation of other lipoproteins followed by timed endpoint, coupled enzymatic methodology	10
Triglycerides (mg/dL)	Timed endpoint, coupled enzymatic methodology	11

*Use of 1 part serum plus 1 part 10% meta-phosphoric acid for Multiple Supp users versus NHANES use of 1 part serum plus 4 part meta-phosphoric acid.

** Calculated: LDL-cholesterol = [total cholesterol - HDL-cholesterol - (triglycerides/5)] Ref = reference, LDL = low-density lipoprotein, HDL = high-density lipoprotein.

### Multivitamin/mineral and non-supplement users from NHANES

We used NHANES (NHANES III and 2001–2001) data as a source of comparison data for multivitamin users and non-users of supplements. NHANES is designed to monitor the health and nutrition status of the US population and participation consisted of an in-home interview and an examination in the NHANES mobile examination unit [6]. Dietary supplement intake was assessed in NHANES by asking participants about their dietary supplement usage patterns in the past 30 days prior to their home interview, including use of any vitamins, minerals, or other dietary supplements. NHANES oversamples certain groups, such as older people and low-income individuals, in order to obtain greater precision in estimates for those groups; sample weights are applied by NHANES to adjust for oversampling and nonresponse. NHANES sample selection involves a stratified multistage probability design involving counties, blocks, and households. Consequently, variance estimates require use of special software making use of NHANES strata and primary sampling units to account for the sampling design.

To match the age and race composition of the Multiple Supp users, we selected from NHANES 2001–2002 participants all of the White men and women, >35 years of age, and free of cancer other than non-melanoma skin cancer, who met certain supplement use criteria. Only non-supplement users and users of supplements containing vitamins and/or minerals were included for the present analysis. We identified 602 NHANES participants who consumed no dietary supplements in the 30 days prior to the home interview; these became the 'No Supp' users. We also identified 176 individuals who consumed a multivitamin/mineral and no other dietary supplements, and did so at least 15 days over the past 30 days. These became the 'Single Supp' users. To obtain the most recent data possible for comparative purposes, RBC folate, and serum ferritin, homocysteine, CRP, and lipid data were obtained from NHANES 2001–2002 [7]. However, serum retinol, ascorbic acid, alpha tocopherol, and serum carotenoid data were not available from NHANES 2001–2002 or 2003–2004. Therefore, data for these variables for Single Supp and No Supp users who were White, >35 years of age, and free of cancer other than non-melanoma skin cancer were obtained from NHANES III, 1988–1994 [8,9]. The geographical distribution of the individuals in the three users groups is not known. In addition, the duration of dietary supplement usage by NHANES participants, aside from the 30 days prior to their home interview, is unknown.

### Statistical analyses

After combining the Multiple Supp users with the Single Supp and No Supp users from NHANES, data were reweighted. The weights for the NHANES groups were calculated by dividing each NHANES weight by the sum of the NHANES weights of the user group. Appropriate weights were used depending on whether the data came from the interview or the Mobile Examination Center. Multiple Supp users were assigned a weight of 1. Strata and primary sampling units from NHANES were used for those user groups. For the Multiple Supp users, a new stratum variable was assigned, and each member of the Multiple Supp group was assigned to a unique primary sampling unit. These adjustments permit more accurate variance estimates and account for stratification factors. However, the Single Supp and No Supp users from NHANES should not be interpreted as being nationally representative samples. Calculations used SUDAAN Version 9.0 (Research Triangle Institute International, Research Triangle Park, North Carolina).

Differences in the characteristics of the supplement user groups were evaluated using chi-square methods for categorical variables and one-way analysis of variance for continuous variables. Multiple regression techniques were employed to examine differences in nutrient and biomarker concentrations of user groups. Comparisons of mean nutrient concentrations among user groups (RBC folate, and serum retinol, ascorbic acid, alpha tocopherol, carotenoids, and ferritin) were adjusted for sex and age. Comparisons of biomarker concentrations among user groups (serum homocysteine, CRP, total cholesterol, and lipids) were further adjusted for age^2^, education category (<high school, high school graduate, or >high school), income category (<$30,000, ≥$30,000 and <$40,000, ≥$40,000 and <$50,000, ≥$50,000 and <$60,000, ≥$60,000 and <$70,000, and ≥$70,000) and income^2^, and body mass index (BMI). For outcomes treated as dichotomous variables, logistic regression methods were used to estimate odds ratios (OR) with 95% confidence intervals (CI), adjusted for sex, age and age^2^, education category, income category and income^2^, and BMI. The model fit was examined using the Hosmer and Lemeshow Goodness-of-Fit test. The referent group for risk estimation was the No Supp user group. Statistical significance was defined as p < 0.05.

Elevated blood pressure was defined as >80 mmHg for diastolic and/or >120 mmHg for systolic blood pressure [13]. Suboptimal and elevated nutrient and biomarker concentrations were defined as: <317 nmol/L RBC folate [14,15]; <37.5 nmol/L and >600 nmol/L for serum 25-hydroxyvitamin D [16-19]; >9 μmol/L for serum homocysteine [20]; >3.0 mg/L for serum CRP [21]; ≥200 mg/dL for serum total cholesterol [22]; <40 mg/dL for serum high-density lipoprotein (HDL)-cholesterol for men and <50 mg/dL for women [22]; ≥130 mg/dL for serum low-density lipoprotein (LDL)-cholesterol [22]; <5 for the ratio of total cholesterol to HDL-cholesterol [22]; ≥150 mg/dL for serum triglycerides [22]; and <0.4 mg/dL and >1.0 mg/dL for ascorbic acid [23,24].

## Results

Significant differences by user group were found for sex, age, BMI, education, and household income (Table 2). The proportion female increased with increasing degree of supplement use. The two supplement user groups were older than the No Supp users, and had lower BMI. Multiple Supp users were well-educated with 86% having greater than a high school education compared to approximately 55% in the other two user groups. The proportion with annual household incomes less than $30,000 was 19.7% and 17.3% in the No Supp and Single Supp users respectively, and 2.5% in the Multiple Supp group. Participants were predominately White because of the racial composition of the Multiple Supp users and the fact that Single Supp and No Supp users were matched to Multiple Supp users on race.

**Table 2:** Characteristics of the three supplement user groups: multiple dietary supplement users (Multiple Supp) from Shaklee, and multivitamin/mineral users (Single Supp) and non-users (No Supp) from NHANES 2001–2002.

Characteristic	No Supp n = 602	Single Supp n = 176	Multiple Supp n = 278	P-value*
Sex (% female)	40.5	44.6	57.9	<0.0001
Race (% white)	100	100	98.9	
Age, years (mean, SE)	53.7 (0.59)	57.0 (0.99)	63.3 (0.48)	<0.001
BMI, kg/m^2 ^(mean, SE)	29.4 (0.32)	27.9 (0.46)	25.9 (0.31)	<0.001
Education (%)				<0.0001
< High School Graduate	16.8	14.7	1.8	
High School Graduate	29.1	30.1	12.2	
> High School Graduate	54.2	55.2	86.0	
				
Annual income (%)				<0.0001
< $30,000	19.7	17.3	2.5	
$30,000–39,999	16.4	14.4	6.8	
$40,000–49,999	18.2	19.9	4.7	
$50,000–59,999	6.9	10.2	13.3	
$60,000–69,999	6.3	3.3	10.8	
≥ $70,000	32.6	34.9	61.9	

*Differences among the supplement user groups were evaluated using chi-square methods for categorical variables and one-way analysis of variance for continuous variables. BMI = body mass index.

### Dietary supplement usage patterns

By definition, No Supp users consumed no supplements and Single Supp users consumed only a multivitamin/mineral dietary supplement and did so approximately every other day or more often. Multiple Supp users reported taking an average of 25 different supplements in the past 12 months (median, 26), and took an average of 17 different supplements every day (median, 18) (data not shown). Dietary supplements consumed on a daily basis by more than 50% of both male and female Multiple Supp users included a multivitamin/mineral, B-complex, vitamin C, vitamin E, calcium with vitamin D, a herbal immune supplement, carotenoids, omega-3 fatty acids, flavonoids, lecithin, coenzyme Q10 with resveratrol, alfalfa, and glucosamine (Table 3). In addition, a majority of women also consumed gamma linolenic acid and a probiotic supplement, whereas men also consumed zinc, garlic, a soy protein supplement, and saw palmetto daily.

**Table 3:** Dietary supplement usage patterns of long-term users of multiple dietary supplements (Multiple Supp users).

Dietary supplement type	Average daily usage	% of women who were daily users	% of men who were daily users
Multivitamin/mineral (with or without iron)	*	89.5	84.7
B-Complex	^†^	87.7	79.5
Vitamin C	1000 mg/d	86.4	80.3
Vitamin E (d-alpha tocopherol and mixed tocopherols)	800 IU/d	79.0	74.4
Carotenoids	^‡^	79.6	74.4
Flavonoids (from a variety of sources)	362 mg/d	86.4	52.1
Calcium with vitamin D	^§^	81.5	68.4
Zinc	15 mg/d	48.2	55.6
Lecithin	3.1 g/d	71.6	66.7
Alfalfa (*Medicago sativa*, leaf)	2.85 g/d	75.9	70.9
Garlic (*Allium sativum*, clove)	1 g/d	49.4	54.7
Omega-3 fatty acids (from fish oil)	1000 mg/d	81.5	73.5
Coenzyme Q10 with resveratrol	^#^	69.8	64.1
Soy protein isolate	14 g/d	46.9	55.6
Glucosamine hydrochloride	1500 mg/d	59.3	57.3
Saw palmetto extract (*Serenoa repens*, berry)	320 mg/d	1.9	67.5
Gamma linolenic acid (from borage seed oil)	180 mg/d	62.4	35.0
Probiotic supplement (from *Bifidobacterium longum *and *Lactobacillus acidophilus*)	^††^	50.0	32.5
Herbal immune supplement	^‡‡^	60.5	58.1

*35–200% of the Daily Value for 25 vitamin and minerals and also includes 5 trace minerals.

^†^200–2700% of the Daily Value for 8 B vitamins.

^‡^5000 IU/d vitamin A as beta carotene, 5 mg/d lycopene, 5 mg/d lutein, 1.5 mg/d alpha carotene, 200 mcg/d zeaxanthin, 400 mcg/d astaxanthin.

^§^1500 mg/d calcium, 600 IU/d vitamin D, 600 mcg/d vitamin K, 600 mg/d magnesium, and <15% of the Daily value for zinc, copper, and manganese.

^#^30 mg/d coenzyme Q10 and 640 mcg/d resveratrol.

^††^250 million/d *Bifidobacterium longum *and 250 million/d *Lactobacillus acidophilus*.

^‡‡^500 mg/d plant extracts from pumpkin seed (*Curcubita moschata*), safflower flower (*Carthamus tinctorius*), asian plantain seed (*Plantago asiatica*), Japanese honeysuckle flower (*Lonicera japonica*).

### Serum nutrient and biomarker concentrations

There was a statistically significant increase in RBC folate and serum concentrations of ascorbic acid, alpha and beta carotene, and alpha tocopherol with degree of supplementation use (Table 4). For these nutrients, Single Supp users had significantly higher nutrient concentrations than No Supp users, and Multiple Supp users had higher concentrations than the Single Supp group. Serum retinol was higher among Single and Multiple Supp users compared to No Supp users. Among women, serum ferritin was highest in Multiple Supp users and lower in the Single Supp users compared to No Supp users. Among men, serum ferritin was significantly lower in the Multiple Supp users compared to the two other supplement groups.

**Table 4:** Serum nutrient and biomarker concentrations of a sample of long-term users of multiple dietary supplements (Multiple Supp), and of multivitaminmineral users (Single Supp) and non-users (No Supp) from NHANES 2001–2002 and NHANES III 1988–1994.

Characteristic	No Supp n = 602	Single Supp N = 176	Multiple Supp N = 278	p-value*
	Mean (SE)			

NUTRIENTS				
RBC folate (nmol/L)^‡^	646.7 (8.9) a	891.1 (30.7) b	1153.4 (11.8) c	<0.001
Retinol (μg/dL)^†^	59.2 (0.5) a	64.3 (0.7) b	65.0 (1.0) b	<0.001
Ascorbic acid (mg/dL)^†^	0.66 (0.02) a	0.94 (0.02) b	1.62 (0.03) c	<0.001
Alpha tocopherol (mg/dL)^†^	1.1 (0.01) a	1.4 (0.02) b	2.9 (0.06) c	<0.001
Alpha carotene (μg/dL)^†^	4.5 (0.1) a	5.9 (0.2) b	27.5 (1.3) c	<0.001
Beta carotene (μg/dL)^†^	18.5 (0.3) a	27.0 (1.2) b	62.7 (2.8) c	<0.001
Ferritin (μg/L)^‡ ^Male	198.2 (6.1) a	205.2 (12.6) a	117.6 (5.9) b	<0.001
Ferritin (μg/L)^‡ ^Female	101.7 (7.1) a	74.9 (8.4) b	117.4 (3.3) c	<0.001
25-hydroxyvitamin D (nmol/L)			131.4 (45.3)^§^	
BIOMARKERS				
Homocysteine (μmol/L)^‡^	9.6 (0.2) a	9.1 (0.5) a	6.1 (0.3) b	<0.001
C-reactive protein (mg/dL)^‡^	0.46 (0.04) a	0.32 (0.03) b	0.19(0.03) c	<0.01
Total cholesterol (mg/dL)^‡^	211.5 (2.9) a	212.1 (3.6) a	203.1 (2.7) b	<0.05
LDL-cholesterol (mg/dL)^‡^	125.1 (2.8)	129.5 (4.2)	122.1 (2.6)	NS (>0.05)
HDL-cholesterol (mg/dL)^‡^	50.9(0.5) a	53.3 (1.4) a	57.5 (1.1) b	<0.001
Ratio total-cholesterol to HDL-cholesterol^‡^	4.5 (0.07) a	4.3 (0.13) a	4.0 (0.09) b	<0.001
Triglycerides (mg/dL)^‡^	180.1 (16.7) a	145.3 (9.4) a	121.0 (6.8) b	< 0.01

*Multiple regression techniques were employed to examine differences in nutrient and biomarker concentrations of user groups. Differences in nutrient concentrations were adjusted for sex and age. Differences in biomarker concentrations were further adjusted for age^2^, education, income and income^2^, and body mass index (BMI). P-values represent the statistical significance for comparisons of user group means with different letters; if these comparisons varied in significance level, the most significant is shown.

^†^Data obtained from NHANES III 1988–1994.

^‡^Data obtained from NHANES 2001–2002.

^§^Comparable 25–hydroxy vitamin D data not available from NHANES.

LDL = low-density lipoprotein, HDL = high-density lipoprotein.

There was a decrease in concentrations of biomarkers associated with disease risk as supplement use increased (Table 4). Across the three user groups there was a decrease in serum CRP and the ratio of total cholesterol to HDL-cholesterol, and an increase in HDL-cholesterol concentration. Serum homocysteine and triglycerides were lower in the Multiple Supp users than in the other two groups. Finally, serum total cholesterol was significantly lower in the Multiple Supp group, whereas LDL-cholesterol concentrations did not vary significantly by supplement use.

Among Multiple Supp users, no individuals had suboptimal or elevated serum 25-hydroxyvitamin D concentrations (range: 39.3–263.8 nmol/L). No comparative 25-hydroxyvitamin D data were available from NHANES. No Multiple Supp users had suboptimal serum ascorbic acid concentrations (<0.4 mg/dL), compared with 9.4% among Single Supp users and 32.4% among the No Supp group (data not shown). Similarly, 94.1% of Multiple Supp users had serum ascorbic acid concentrations >1.0 mg/dL, compared with 46.6% of Single Supp users and 21.9% of No Supp users (data not shown).

### Blood pressure and adverse biomarker concentrations

Adjusted risk estimates for elevated blood pressure and suboptimal or elevated concentrations of serum biomarkers are reported in Table 5. Compared to No Supp users, Multiple Supp users had significantly reduced risks of elevated serum homocysteine, triglycerides, and ratio of total cholesterol to HDL-cholesterol, and significantly reduced risk of low HDL-cholesterol. For elevated serum CRP, the risk among Multiple Supp users could not be estimated because of the absence of any persons with elevated values of CRP. Multiple Supp users also had a significantly reduced risk of elevated blood pressure compared to No Supp users. Single Supp users had significantly lower risks of elevated serum homocysteine, and low serum HDL-cholesterol. Risks of elevated serum total cholesterol and LDL-cholesterol did not significantly differ across the supplement user groups.

**Table 5:** Risk of suboptimal or elevated serum biomarker concentrations and elevated blood pressure by supplement user group.

Outcome	Cases: n/(%)	OR*	95% CI*
Homocysteine (>9 μmol/L)			
Multiple Supp users	30/(10.9)	0.11	0.06–0.19
Single Supp users	69/(37.0)	0.53	0.34–0.83
No Supp users	282/(45.3)	1.00	
C-reactive protein (>0.3 mg/dL)			
Multiple Supp users	0/(0.00)	^†^	^†^
Single Supp users	51/(30.6)	0.66	0.40–1.09
No Supp users	230/(41.3)	1.00	
Total cholesterol (>200 mg/dL)			
Multiple Supp users	145/(52.5)	0.78	0.56–1.09
Single Supp users	89/(57.8)	1.08	0.72–1.62
No Supp users	291/(54.3)	1.00	
HDL-cholesterol (<40 mg/dL males, <50 mg/dL females)			
Multiple Supp users	44/(15.8)	0.50	0.33–0.75
Single Supp users	40/(24.5)	0.58	0.41–0.83
No Supp users	203/(38.0)	1.00	
Ratio total cholesterol to HDL-cholesterol (<5)			
Multiple Supp users	35/(12.7)	0.49	0.32–0.74
Single Supp users	48/(35.3)	1.05	0.64–1.73
No Supp users	189/(36.5)	1.00	
Triglycerides (≥150 mg/dL)			
Multiple Supp users	52/(18.8)	0.44	0.27–0.71
Single Supp users	31/(41.5)	0.99	0.50–1.94
No Supp users	157/(42.8)	1.00	
LDL-cholesterol (>130 mg/dL)			
Multiple Supp users	109/(39.5)	0.96	0.56–1.65
Single Supp users	32/(47.6)	1.19	0.70–2.03
No Supp users	101/(42.7)	1.00	
Blood Pressure (DBP>80 mmHg or SBP>120 mmHg)			
Multiple Supp users	154/(55.6)	0.61	0.41–0.92
Single Supp users	117/(71.2)	1.34	0.88–2.03
No Supp users	353/(64.6)	1.00	

* Logistic regression methods were used to estimate odds ratios (OR) with 95% confidence intervals (CI), adjusted as in footnote to Table 4. The referent group for risk estimation was the No Supp group.

^†^OR and 95% CI not estimable because zero events in the user group. Number and percent shown instead.

HDL = high-density lipoprotein, LDL = low-density lipoprotein, DBP = diastolic blood pressure, SBP = systolic blood pressure.

### Health status and disease prevalence

Prevalence of self-reported disease was low in each user group and most risk estimates did not reach statistical significance (Table 6). An exception was the reduced risk of diabetes in Multiple Supp users (OR = 0.27; 95% CI: 0.12–0.59). Risk of coronary heart disease was also lower in this group (OR = 0.48; 95% CI: 0.22–1.09), but was only marginally statistically significant. For self-assessed health status, Multiple Supp users, but not Single Supp users, were less likely to report their health as 'good, fair, or poor' versus 'excellent or very good' (OR = 0.26; 95% CI: 0.17–0.41) compared to the No Supp users group (Table 6).

**Table 6:** Risk of prevalent disease, by supplement user group.

Outcome	Cases: n/(%) **	OR*	95% CI*
Diabetes			
Multiple Supp users	8/(2.9)	0.27	0.12–0.59
Single Supp users	23/(11.1)	1.11	0.74–1.67
No Supp users	70/(8.5)	1.00	
Coronary Heart Disease			
Multiple Supp users	14/(5.0)	0.48	0.22–1.09
Single Supp users	17/(7.1)	1.08	0.52–2.25
No Supp users	47/(5.6)	1.00	
Heart Attack			
Multiple Supp users	7/(2.5)	0.51	0.18–1.41
Single Supp users	16/(7.6)	1.76	0.90–3.42
No Supp users	44/(4.4)	1.00	
Angina			
Multiple Supp users	6/(2.2)	0.41	0.14–1.18
Single Supp users	12/(6.8)	1.71	0.82–3.59
No Supp users	35/(4.7)	1.00	
Congestive Heart Failure			
Multiple Supp users	4/(1.4)	0.73	0.18–2.95
Single Supp users	9/(2.8)	0.88	0.26–2.97
No Supp users	31/(3.5)	1.00	
Stroke			
Multiple Supp users	5/(1.8)	1.42	0.39–5.17
Single Supp users	6/(2.6)	1.26	0.34–4.67
No Supp users	29/(2.5)	1.00	
Arthritis			
Multiple Supp users	88/(31.7)	1.00	0.69–1.46
Single Supp users	71/(31.8)	1.23	0.77–1.98
No Supp users	189/(26.7)	1.00	
Emphysema			
Multiple Supp users	3/(1.1)	0.55	0.09–3.41
Single Supp users	4/(1.8)	0.73	0.32–1.64
No Supp users	23/(2.3)	1.00	
Health Status^†^			
Multiple Supp users	44/(15.8)	0.26	0.17–0.41
Single Supp users	86/(44.4)	0.75	0.45–1.23
No Supp users	302/(50.9)	1.00	

* Logistic regression methods were used to estimate odds ratios (OR) with 95% confidence intervals (CI), adjusted for sex, age and age^2^, education, income and income^2^, and body mass index. The referent group for risk estimation was the No Supp group.

** Number of events, weighted percent

^†^Self-assessed as 'good, fair, or poor' versus 'excellent or very good.'

## Discussion

Little has been reported about daily users of multiple dietary supplements. We assembled a sample of long-term users of multiple dietary supplements for the purpose of describing their supplement usage patterns. On average, Multiple Supp users in this study consumed 17 different supplements every day. Dietary supplements consumed on a daily basis by more than 50% of Multiple Supp users included a multivitamin/mineral, B-complex, vitamin C, carotenoids, vitamin E, calcium with vitamin D, omega-3 fatty acids, flavonoids, lecithin, alfalfa, coenzyme Q10 with resveratrol, glucosamine, and a herbal immune supplement. Women also consumed gamma linolenic acid and a probiotic supplement, whereas men also consumed zinc, garlic, a soy protein supplement, and saw palmetto (Table 2). By comparison, in NHANES 1999–2000, 52% of those surveyed reported taking a dietary supplement in the past 30 days; 35% reported having taken a multivitamin/mineral; 12.7% having taken vitamin E; 12.4% having taken vitamin C; 10.4% having taken calcium; and 5.2% having taken B-complex vitamins [4]. Consistent with other reports describing dietary supplement users [4], Multiple Supp users as a group were primarily White, better educated, more affluent, and older.

A second objective of the study was to conduct a cross-sectional comparison of the relevant biomarker and nutrient concentrations in the Multiple Supp users, with those of Single Supp users (multivitamin/mineral users) and No Supp users (non-users) in NHANES 2001–20002 and NHANES III. Nutrient concentrations in serum or RBC generally increased across the three supplement user groups. It is notable that Single Supp users had higher circulating concentrations than No Supp users for RBC folate and serum retinol, ascorbic acid, and alpha tocopherol. This fact has important implications for nutrition intervention trials, some of which have permitted participants, including those in the control group, to take a multivitamin/mineral supplement [25]. Our findings suggest that this practice may weaken the power of trials to detect treatment effects of the study supplement.

Degree of supplement use was associated with several markers of disease risk. Elevated serum homocysteine concentrations were found in approximately 45% of No Supp users, 37% of Single Supp users, and 11% of Multiple Supp users, despite the fact that the data were obtained after the 1998 fortification of grain products with folate, and despite the low prevalence of inadequate RBC folate in any of the user groups. If moderately-elevated serum homocysteine is shown to be an important cardiovascular disease risk factor, the findings of this study suggest that intake of the relevant B vitamins above what is typically found in a multivitamin/mineral supplement, and/or the diet, may produce a more favorable homocysteine-lowering effect.

Circulating CRP concentration has been shown to be predictive of future cardiovascular disease risk in prospective studies among asymptomatic individuals and may have a direct effect on the progression of atherosclerosis [26]. In the present study, serum CRP concentrations decreased and serum ascorbic acid increased with increasing degree of supplement use (Tables 2 and 4). This finding is consistent with recent reports that vitamin C supplementation lowers plasma CRP concentrations [27], and that dietary intakes of vitamin C and plasma ascorbic acid concentration are inversely associated with plasma CRP [28]. Studies are underway to confirm the impact of vitamin C supplementation on circulating CRP and other markers of immune function.

Serum ascorbic acid concentration >1.0 mg/dL has been suggested as optimal relative to reduced risk of cardiovascular disease and cancer [24]. By this definition, over 94% of Multiple Supp had optimal concentrations, compared to approximately 47% and 22% in Single Supp and No Supp users, respectively. Suboptimal concentrations, defined as <0.4 mg/dL [23], were not found in the Multiple Supp user group, and were found in approximately 9% and 32% of Single Supp and No Supp users, respectively.

Serum 25-hydroxyvitamin D in Multiple Supp users is of interest because of possible beneficial and adverse effects from supplemental intake of vitamin D. Improving 25-hydroxyvitamin D concentrations above the level associated with subclinical deficiency (<37.5 nmol/L) may reduce the risk of skeletal fractures [16-19]. Also, parathyroid hormone concentrations become minimal when 25-hydroxyvitamin D concentrations exceed 100 nmol/L. Conversely, serum concentrations >600 nmol/L are associated with hypercalcemia [19]. Among Multiple Supp users, no individuals had serum 25-hydroxyvitamin D concentrations <37.5 nmol/L or >600 nmol/L, and the mean (SD) was 131.4 (45.26) nmol/L with a range of 39.30 to 263.80 nmol/L. No comparative data were available from NHANES participants.

Supplement use was also associated with lower serum triglycerides and higher HDL-cholesterol concentrations. In consequence, the risk of an elevated ratio of total cholesterol to HDL-cholesterol was significantly lower in the Multiple Supp group compared to the No Supp group (Table 5). Prevalent use of fish oil supplements in the Multiple Supp users may explain these findings as omega-3 fatty acids in fish oil have been shown to increase HDL-cholesterol and decrease triglyceride concentrations [29]. Although beyond the scope of the present study, lower risk of cardiac arrhythmias may be another benefit of consumption of omega-3 fatty acids [30,31].

Self-assessed health status has been found to be a remarkably good marker of prospective health outcomes [32]. Compared to No Supp users, Multiple Supp users were more likely to describe their health as 'very good' or 'excellent', whereas this was not the case with Single Supp users (Table 6).

Risk of elevated systolic or diastolic blood pressure was significantly lower in the Multiple Supp group compared to No Supp users, but not in Single Supp users compared to No Supp users (Table 5). Dietary interventions have been shown to reduce blood pressure [33]. However, clinical trials of individual nutrients have typically found small and inconsistent effects [33]. Dietary folate and vitamin C, as well as plasma ascorbic acid, have been found to be inversely associated with blood pressure in observational studies [34,35]. However, intervention trials with vitamin C have yielded inconsistent results [35], while in two small trials, folic acid has been effective at lowering blood pressure [36,37]. The finding of a lower risk of elevated blood pressure in the Multiple Supp group, which also had the highest concentrations of RBC folate and serum ascorbic acid, suggests that the relationship between these nutrients and blood pressure may warrant further investigation.

The lower risk of diabetes in the Multiple Supp group is consistent with evidence that oxidative stress may be a mechanism linking insulin resistance with dysfunction of pancreatic beta cells and endothelial dysfunction, eventually leading to diabetes [38]. Although biomarkers of oxidative stress were not available in the three user groups, serum concentrations of antioxidants including ascorbic acid, carotenoids, and alpha tocopherol were all significantly higher in the Multiple Supp users compared to the other groups (Table 2).

An important limitation of the study is the fact that the data are cross-sectional, and therefore the reported associations, particularly with health outcomes (i.e., blood pressure and diabetes), cannot presume causality. Also, the three user groups should not be interpreted to represent unbiased national estimates. In addition, although we adjusted for potential confounders such as age, sex, income, education, and BMI, residual confounding could possibly account for the findings. Better access to health care and variables related to higher socioeconomic status in the supplement user groups (e.g., healthier diets and lifestyles) are logical hypotheses that may account for these results. However, the No Supp and Single Supp user groups were similar with respect to education and income (Table 2), so these factors may not explain the significantly better nutrient and C-reactive protein concentrations seen for Single Supp users in Table 4, and homocysteine and HDL risk levels (Table 5). Furthermore, the lack of differences across the three user groups in biomarkers such as serum total cholesterol and LDL-cholesterol suggest that factors related to health care access and socioeconomic status do not fully explain the results.

This study is the first we are aware of to describe the usage patterns of long-term users of multiple dietary supplements, an unusual sample that cannot be captured in national representative surveys. While only 3 individuals out of over 11,000 surveyed in NHANES 2001–2002 had taken 20 or more kinds of dietary supplements in the past 30 days, in our Multiple Supp users, 87% of the sample reported having taken 20 or more kinds of supplements daily. Thus, this sample of Multiple Supp users affords a rare opportunity to understand a segment of the population that take multiple dietary supplements, and to understand the nutrient and other correlates of this practice. In addition, Multiple Supp users were long-term consumers of dietary supplements. Thus, their serum nutrient and biomarker concentrations were likely to be representative of their long-term supplementation patterns.

## Conclusion

This sample of long-term users of multiple dietary supplements was found to consume a broad array of vitamin/mineral, herbal, and condition-specific dietary supplements on a daily basis. They were more likely to have optimal concentrations of biomarkers associated with reduced disease risk, and less likely to have suboptimal circulating nutrient concentrations, elevated blood pressure, and diabetes than multivitamin/mineral users and non-users. There was no evidence that intake of vitamin D in multiple supplement users was excessive based on serum 25-hydroxyvitamin D concentrations. These findings should be confirmed by studying the dietary supplement usage patterns and health and nutrition status of other groups of heavy users of dietary supplements. Our study findings should also be weighed in the context of recent randomized controlled trials and related meta-analyses [39,40] which have raised concern about potential detrimental effects of select dietary supplements, particularly beta carotene and alpha tocopherol. While the typical daily intake of beta carotene from supplements in the Multiple Supp group was low relative to those studies (approximately 5000 IU/d of vitamin A as beta carotene), the typical intake of alpha tocopherol was 800 IU/d, similar to the dose in a number of randomized trials. Further research on usage patterns of dietary supplements and the potential health effects of dietary supplements is needed. The present report serves as a baseline to which the findings of subsequent monitoring efforts can be compared.

## Abbreviations

US = United States, NHANES = National Health and Nutrition Examination Survey, RBC = red blood cell, CRP = C-reactive protein, BMI = body mass index, OR = odds ratio, CI = confidence interval, HDL = high-density lipoprotein, LDL = low-density lipoprotein.

## Competing interests

The study was financially supported by Shaklee Corporation. LGW and JFM are employed by, and CDJ is a scientific consultant to the sponsor. GB has received research funding from the sponsor.

## Authors' contributions

GB, CDJ, and LGW participated in the design of the study, acquisition of the data, analysis and interpretation of the data, and drafting and revising of the manuscript.

EPN and JFM participated in the acquisition of the data and revising of the manuscript. TBD and MLH participated in the analysis and interpretation of the data and drafting and revising of the manuscript. All authors read and approved the final manuscript.
